# Prevalence and Risk Factors of Internet Addiction among Hungarian High School Teachers

**DOI:** 10.3390/life11030194

**Published:** 2021-03-03

**Authors:** Gábor Tóth, Krisztian Kapus, David Hesszenberger, Marietta Pohl, Gábor Kósa, Julianna Kiss, Gabriella Pusch, Éva Fejes, Antal Tibold, Gergely Feher

**Affiliations:** 1 Centre for Occupational Medicine, Medical School, University of Pécs, 7624 Pécs, Hungary; titkarsag@bajakorhaz.hu (G.T.); kapus.krisztian@kozut.hu (K.K.); pohlmarietta@bmvk.hu (M.P.); kosagabor@kosagabor.hu (G.K.); julianna.kiss@shl.hu (J.K.); titkarsag@komloikorhaz.hu (É.F.); tibold.antal@pte.hu (A.T.); 2 Szent Rókus Hospital, 6500 Baja, Hungary; 3 Department of Laboratory Medicine, Medical School, University of Pecs, 7624 Pécs, Hungary; david.hesszenberger@aok.pte.hu; 4 Department of Neurology, Medical School, University of Pecs, 7624 Pécs, Hungary; fmk@pte.hu; 5 Hospital of Komlo, 7300 Komlo, Hungary; 6 Neurology Outpatient Clinic, EÜ-MED KFT, 7300 Komló, Hungary

**Keywords:** internet addiction, adult prevalence, risk factor, teacher

## Abstract

The extensive availability of internet has led to the the recognition of problematic internet use (so called internet addiction, IA) mostly involving adolescents. There is limited data about the prevalence of IA in adults. Here we present a study focusing on the prevalence and risk factors of internet addiction among high school teachers. Overall 2500 paper-based questionnaires were successfully delivered and 1817 responses received (response rate of 72.7%). In our study 1194 females (65.7%) and 623 males (34.3%) participated. In a multivariate analysis including of all factors (demographic data, internet habits, comorbidity etc.) age <35 years (OR: 6.098, CI: 5.09–7.08, *p* < 0.001), male gender (OR = 5.413, CI: 4.39–6.18, *p* = 0.002), surfing on the internet > 5 h daily (OR 2.568, CI: 2.03–3.39, *p* < 0.001), having no children (OR: 1.353, CI: 1.13–1.99, *p* = 0.0248), and having secondary employment (OR = 11.377, CI: 8.67–13.07, *p* = 0.001) were significantly associated with internet addiction. This is the first study from Hungary showing the prevalence and risk factors of internet addiction among high school teachers. A small, but significant proportion suffered from IA. Our study also draws attention to the risk factors of IA such as younger age, family status and working type.

## 1. Introduction

The widespread use of internet has dramatically changed our lives by the 21st century. Although this technological revolution has improved many aspects of our lives and it is now an essential part of the everyday routine, including work, private life and social functioning, many studies reported the misuse of internet (problematic internet use, internet addiction; IA) as summarized in a recent meta-analysis including 113 epidemiologic studies covering 693,306 subjects showed that the pooled prevalence of internet addiction was 7.02% (95% CI, 6.09–8.08%) in the population aged 7–60 years [1]. Age (adolescent samples), lower cut-off scores (and type of questionnaire), sample size (more common is smaller samples), and country of origin (more frequent in Asian studies) were significant predictors of higher prevalence [2,3].

The individual suffering from internet addiction may be not aware of it and the symptoms remain unrecognized by his or her relatives, friends and colleagues [4]. 

IA may be classified as a compulsive-impulsive spectrum disorder based on symptomatology, but it has been under considerable research, and is not included in the recently published 5th edition of the Diagnostic and Statistical Manual DSM-V [5,6].

IA seems to have several risk factors such as younger age at the start of internet use, male gender, daily time interval, goal of internet use and low socioeconomic status [5,7]. Psychosocial factors such as low self-concept and and lack of family support are also associated with problematic internet use [8,9]. Problematic internet use seems to be associated with medical conditions such as anxiety, depression, drug abuse and malnutrition [10,11]. 

IA is mainly studied in adolescents aged 14–18 (who may be at heightened risk for mental health problems), raising the possibility of school based prevention [12,13]. Their teachers also have increased opportunities to use the internet. Therefore we targeted high-school teachers who are amongst the first lines of prevention.

The aim of our research was to detect the prevalence of internet addiction among high school teachers and its risk factors including age, gender, family type, working years, daily internet use, and the goal of being online. Medical conditions may be associated with IA such as smoking, alcohol and drug intake, hypertension, diabetes, ischemic heart disease, musculoskeletal pain, and history of depression were also recorded.

## 2. Materials and Methods

This prospective, cross-sectional, paper-based questionnaire study was conducted between January 2020 and August 2020 in 14 sites in Hungary. The names of the included schools are mentioned in the Acknowledgement part. 

The study was approved by the Ethical Committee of the University of Pecs (8434-PTE 2020). Consent was obtained from the individuals prior to data collection. Paper-based questionnaires were delivered to those who had previously agreed to participate by signing an informed consent.

Inclusion criteria were working as a high school teacher, being between 18 and 65 years of age and being employed at the time of the study apart from the type of employment (public servant, subcontractor, etc.).

Exclusion criteria were being under 18 or over 65 years of age, being on permanent leave or refusing to participate in the study.

Demographic criteria included age, gender, marital status, number of children, type of work, years spent with work, work schedule, legal relation, and secondary employment.

Included risk factors and medical conditions were smoking, alcohol and illicit drug intake; diabetes, hypertension, ischemic heart disease, musculoskeletal pain, and history of depression.

As there are no clear diagnostic criteria for the internet addiction it is highly recommended to measure excessive internet use with a continuous questionnaire [4]. We chose the problematic internet use questionnaire (PIUQ) because its structure tightly adheres to the proposed diagnostic criteria for internet addiction and was created based on the clinimetric and psychometric analysis of Young’s internet addiction test independently validated by several groups and used in our previous published work [14,15,16,17]. The questionnaire contains 18 items, each scored on a 5-point Likert-type scale ranging from 1 (never) to 5 (always). A confirmatory factor analysis verified the three factor model of the questionnaire, each subscale contains six items. Obsession subscale refers to obsessive thinking about the internet (daydreaming, rumination, and fantasizing) and withdrawal symptoms caused by the lack of internet use (anxiety and depression) (“How often do you feel tense, irritated, or stressed if you cannot use the Internet for as long as you want to?”). Neglect subscale contains items about neglecting everyday activities, social life, and essential needs (“How often do you spend time online when you’d rather sleep?”). Control disorder subscale reflects difficulties in controlling time spent on the Internet (“How often do you realize saying when you are online, “just a couple of more minutes and I will stop”?”). Since in this study we focused on global psychological consequences of internet addiction, we used PIUQ total score in statistical analyses, which was computed by summing the scores on all the items of the scale. A total score exceeding 41 points suggests internet addiction [15].

Data were evaluated as means ± SD (standard deviation) by Student’s *t*-test, the chi square test and the Pearson’s rank-order correlation. Logistic regression analysis was used to determine the significance of the different parameters as independent risk factors of IA. The analysis was performed with appropriate adjustments for differences in risk factors and medication usage. For all odds ratios, an exact CI of 95% was constructed in our study. Data analysis was performed using SPSS (version 22.0, IBM, New York, NY, USA).

## 3. Results

### 3.1. Baseline Characteristics

Overall 2500 paper-based questionnaires were successfully delivered and 1817 responses received (response rate of 72.7%). 

In our study 1194 females (65.7%) and 623 males (34.3%) participated. Age distribution was the following: 18–25 years 2.5% (46/1817), 26-35 years 11.9% (217/1817), 36–45 years 31.8% (577/1817), 46–55 years 33.1% (602/1817), 56–62 years 15.7% (285/1817), and 5.0% above 62 (90/1817) (Table 1)

Of the participants, 73.6% (1339/1817) were married or lived in a relationship, 26.4% (478/1817) were single. 23.1% (419/1817) had no children, 22.8% (414/1817) had one child, 38.9% (706/1817) had two, and 15.2% (278/1817) had three or more children. 

Of the participants, 0.5% (9/1817) had elementary degree, 5.8% (105/1817) had secondary education, and 93.7% (1703/1817) had university graduation. 

Of the participants, 2.9% (54/1817) have been employed for less than a year. 37.0% (671/1817) of the study population have been working between 21 and 40 years, 32.1% (584/1817) have been working between 11 and 20 years, and 2.2% (40/1817) more than 40 years. 12.8% (233/1817) had also a secondary employment (Table 1).

### 3.2. Risk Factors and Previous Diseases

Of the participants, 15.1% (275/1817) were regular smokers, 5.1% (93/1817) were taking alcohol, and 2.9% (52/1817) were taking illicit drugs more or less regularly. 

Of the participants, 22.8% (414/1817) had hypertension, 7.4% (135/1817) were diabetic, 10.2% had ischemic heart disease (186/1817), 8.0% (146/1817) suffered from musculoskeletal pain, and 1.5% (27/1817) had a history of depression (Table 2). 

### 3.3. Duration and Goal of Internet Use

Of the participants, 38.3% (696/1817) spent less than one hour online and 2.0% (35/1817) used the internet more than six hours a day. More than half of the examined workers preferred being online between 6 and 9 p.m. (51.9%, 943/1817). The main goals of internet surfing were to every day work 93.0% (1689/1817), visit community portals 42.5% (773/1817), and listening to music in 30.0% (539/1817). Detailed data can be seen in Table 2.

### 3.4. Internet Addiction

Internet addiction was detected in 5.2% (95/1817) based on the problematic internet use questionnaire. Internet addiction was more common in males (62.1 vs. 32.7%, *p* = 0.001) and workers below 35 years of age (29.5 vs. 13.6%, *p* < 0.001). Being middle-aged or older was protective against IA (54.8 vs. 34.7%, *p* = 0.001, mostly driven by ages between 45 and 55).

IA was more prevalent among singles (23.1 vs. 14%%, *p* = 0.011) and childless (34.7 vs. 22.4%, *p* = 0.004) (Table 3). Living in a relationship (74.2 vs. 63.1%, *p* = 0.018, mostly driven by being married) or having at least two children (55 vs. 40%, *p* = 0.004, mostly driven by having two children) were protective against IA. 

Lower educational level was also associated with IA (3.2 vs. 0.3%, *p* < 0.001).

Working for less than a year was a predictor of IA (7.3 vs. 2.7%, *p* = 0.009), while working for > 20 years was protective (40.1 vs. 21.1%, *p* < 0.001) (Table 3). Having a secondary employment was significantly associated with problematic internet use (85.3 vs. 12.7%, *p* < 0.001).

There was a significant association between the duration of being online and being addicted to the internet (r = 0.36, *p* < 0.001) (Table 4). The cut-off of spending 5 h or more online predicted IA. We found no association between the daily time interval of internet use and IA.

Among the types of internet services internet gaming (25.2 vs. 7.8%), chatting (40 vs. 21.6%), and matchmaking (11.6 vs. 2.4%, *p* < 0.001 in all cases) were significantly associated with IA (Table 4). 

Internet addiction was more prevalent among smokers (34.7 vs. 14.1%), alcohol, and drug users (17.9% vs. 4.4%, 15.8% vs. 2.1%, *p* < 0.001 in all cases). Diabetes (13.7 vs. 7.1%, *p* = 0.016) and history of depression (8.4 vs. 1.1%, *p* < 0.001) were significantly associated with problematic internet use (Table 4).

In a multivariate analysis including all factors (demographic data, internet habits, comorbidity, etc.), age < 35 years (OR: 6.098, CI: 5.09–7.08, *p* < 0.001), male gender (OR = 5.413, CI: 4.39–6.18, p = 0.002), surfing on the internet > 5 h daily (OR 2.568, CI: 2.03–3.39, *p* < 0.001), having no children (OR: 1.353, CI: 1.13–1.99, *p* = 0.0248), and having secondary employment (OR = 11.377, CI: 8.67–13.07, *p* = 0.001) were significantly associated with internet addiction (Table 5).

## 4. Discussion

Internet addiction is a well known phenomena among adolescents, but only few studies focused on its prevalence among adults, and we found only one study including (their) teachers [12].

In a recent meta analysis the rate of internet addiction was 7.2% in the general population, which is far lower than it would be expected by the IA rate of adolescents, which can be as high as 20% [1,18]. In out study the rate of IA was 5.2%, which is comparable to the above mentioned findings.

Internet addiction was more common in males in our study and confirmed the hypothesis of gender-related differences in this addictive behavior [19]. Internet addiction was also more prevalent entrants up to the age of 35 underlying the importance lower age of first internet use and the protective role of increasing age [20,21]. In a multivariate analysis younger age and male gender remained significant predictors of IA.

Singles, childless ones are at higher risk of IA based on our study. The protective role of living in a relationship and having children (and increasing age) was also reported by a previous study [22]. Having no children remained a significant predictor of IA in a multivariate analysis.

Problematic internet use was associated with chatting, gaming, and watching movies taking the types of internet use into account. This is in concordance with recent results showing different distribution patterns of IA based on sex and specific internet services, chatting, gaming, and watching movies (mostly pornography) were strongly associated with this phenomena [23]. Males overwhelmingly use the internet for gaming, while females are mostly involved in blogging and messenger/chatting, which might be related to well-established evidence that females are more interpersonally oriented, while males are more information/task oriented [23]. However, the goal of internet use lost its predictive value in a multivariate analysis. 

The greater internet use was associated with increased prevalence of addiction, previous studies showed being >2 h online as the predecessor of addiction [24,25]. We found the cut-off value of 5 h or more daily internet use as an independent risk factor of IA, which is in concordance with very recent results [25].

Internet addicts reported night time as the preferred time of internet use underlying the addictive nature of the phenomena among adolescents [26]. Bedtime and wake-up time internet use on weekdays and holidays were also significantly associated with later problematic internet use/gaming disorder in children [27]. Our study could not confirm the role of the daily time interval as a risk factor of IA among teachers.

IA was also associated with substance abuse such as alcohol or drugs and a history of depression. The association of IA and psychiatric symptoms is not well understood. An underlying psychopathology (history of addiction) may precipitate internet addiction or IA may lead to the onset of consequent behavioral abnormalities and mood disorders, and finally they may enhance each other [28]. Drug addicts who use stimulants may have specific vulnerability to IA based on a recent study [29].

Diabetes was also significantly associated with internet addiction. Based on a recent meta-analysis each additional 1 h/d of internet use was associated with 8% increased odds of overweight and obesity, which can lead to metabolic syndrome, diabetes, and cardiovascular morbidity [30]. However, a multivariate analysis could not confirm the predictive roles of substance abuse, depression, and diabetes as risk factors of IA.

Secondary employment was also associated with IA and also remained a significant predictor in a multivariate analysis. Being overwhelmed with work can lead to distress and social anxiety, which seem to be predecessors of problematic internet use [26]. Furthermore, overwork can be the predictor of burnout and a recent study showed the possible association of burnout and internet addiction in adolescents [31].

IA has been under considerable research, and has generated controversy, debate, and quarreling among expert researchers, healthcare, and non-healthcare professionals due to insufficient data, poor quality research, and the lack of randomized studies [1]. However, internet addiction seems to be more than just the consequence of mental instability of adolescents. It can be associated with atrophy in the prefrontal and striatal areas similar to other type of addictions and can be related to psychiatric diseases, overweight (metabolic syndrome/diabetes), and chronic musculoskeletal pain [29,31,32]. Furthermore, pooled estimate of the persistence can be as high as 50% [33]. Our study confirmed the presence of internet addiction among adults and its association with some of the above mentioned medical conditions underlying the importance of recognition to avoid further complications.

In summary, this is the first study from Hungary showing the prevalence and risk factors of internet addiction among high school teachers. A small, but significant proportion suffered from IA. Our study also draws attention to the risk factors of IA such as younger age, family status, and working type.

Finally, our article has some limitations. Although it was a prospective study in nature including more than 1700 teachers, it was not representative of internet addiction in the general/adult population. As it was a questionnaire based survey, physical examination was not carried out and we had no detailed information about the medical history of the study population such as type and duration of medical disorders. The above mentioned limitations may influence our findings. Additionally, finally, follow-up was not carried out.

## Figures and Tables

**Table 1:** Baseline characteristics of the study population (N = 1817).

**Gender**	
Female	1194 (65.7%)
Male	623 (34.3%)
**Age**	
18–25 years	46 (2.5%)
26–35 years	217(11.9%)
36–45 years	577 (31.8%)
46–55 years	602 (33.1%)
56–62 years	285 (15.7%)
more than 62 years	90 (5.0%)
**Marital Status**	
single	263 (14.5%)
relationship	257 (14.1%)
married	1082 (59.5%)
divorced/widow	215 (11.9%)
**Number of Children**	
have no children	419 (23.1%)
1 child	414 (22.8%)
2 children	706 (38.9%)
more than 3 children	278 (15.2%)
**Work Schedule**	
regular	1735 (95.5%)
shifts	82 (4.5%)
**Graduation**	
elementary	9 (0.5%)
secondary education	105 (5.8%)
higher education	1703 (93.7%)
** Years Spent with Work **	
1–12 months	54 (2.9%)
1–5 years	205 (11.3%)
6–10 years	263 (14.5%)
11–20 years	584 (32.1%)
21–30 years	383 (21.1%)
31–40 years	288 (15.9%)
more than 40 years	40 (2.2%)
**Secondary Employment**	
no	1584 (87.2%)
yes	233 (12.8%)

**Table 2:** Concomitant diseases, substance abuse, and internet use in the study population.

**Concomitant Diseases (%)**	
taking any medications regularly	495 (27.2%)
smoker	275 (15.1%)
taking alcohol	93 (5.1%)
taking drugs	52 (2.9%)
diabetes	135 (7.4%)
hypertension	414 (22.8%)
cardiovascular disease	186 (10.2%)
musculoskeletal pain	146 (8.0%)
history of depression	27 (1.5%)
**Daily Internet Use (Approximately) (%)**	
1 h	696 (38.3%)
2 h	569 (31.3%)
3 h	287 (15.8%)
4 h	132 (7.9%)
5 h	54 (2.9%)
6 h	44 (2.4%)
>6 h	35 (2.0%)
**Daily Time Interval of Internet Use (Multiply Answer) (%)**	
between 0 and 3 a.m.	186 (10.2%)
between 3 and 6 a.m.	75 (4.1%)
between 6 and 9 a.m.	233 (12.8%)
between 9 and 12 a.m.	349 (19.2%)
12–3 p.m.	209 (11.5%)
3–6 p.m.	441 (24.3%)
6–9 p.m.	943 (51.9%)
9–12 p.m.	357 (19.6%)
**Goal of internet use** **(multiply answer) (%)**	
learning/working	1689 (93.0%)
internet gaming	159 (8.7%)
chat	410 (22.6%)
community portal (Facebook, Twitter, etc.)	773 (42.5%)
matchmaking	52 (2.9%)
movies	328 (18.1%)
music	539 (30.0%)
other	196 (10.8%)

**Table 3:** Comparison of baseline characteristics of the study subgroups. ** *p* < 0.001; * *p* <0.05.

	Not Addicted to Internet (*n* = 1722)	Internet Addiction (*n* = 95)
**Gender**		
Male	**564 (32.7%)**	59 (62.1%) *
Female	1158 (67.2%)	36 (37.9%)
**Age (Years)**		
18–25 years	**39 (2.3%)**	7 (7.4%) *
26–35 years	**196 (11.4%)**	21 (22.1%) *
36–45 years	543 (31.5%)	34 (35.8%)
46–55 years	**585 (34%)**	17 (17.9%) *
56–62 years	273 (15.8%)	12 (12.6%)
more than 62 years	86 (5%)	4 (4.2%)
**Marital Status (%)**		
single	**241 (14%)**	22 (23.1%) *
relationship	240 (14%)	17 (17.9%)
married	**1037 (60.2%)**	43 (45.3%) *
divorced / widow	202(11.7%)	13 (13.7%)
**Number of Children**		
having no children	**386 (22.4%)**	33 (34.7%) *
1 child	390 (22.6%)	24 (25.3%)
2 children	**683 (39.7%)**	23 (24.2%) *
more than 3 children	263 (15.3%)	15 (15.8%)
**Work Schedule**		
regular	1643 (95.4%)	92 (96.8%)
shifts	79 (4.6%)	3 (3.2%)
**Graduation**		
elementary	**6 (0.3%)**	3 (3.2%) **
secondary education	108 (6.3%)	7 (7.4%)
higher education	1618 (96.9%)	85 (89.5%)
** Years Spent with Work **		
1–12 months	**47 (2.7%)**	7 (7.3%) *
1–5 years	191 (11.1%)	14 (14.7%)
6–10 years	246 (14.3%)	17 (17.9%)
11–20 years	547 (31.8%)	37 (38.9%)
21–30 years	**373 (21.7%)**	10 (10.5%) *
31–40 years	**281 (16.3%)**	7 (7.4%) *
more than 40 years	37 (2.1%)	3 (3.2%)
**Secondary Employment**		
no	1503 (87.3%)	14 (14.7%)
** yes **	**219 (12.7%)**	81 (85.3%) **

**Table 4:** Comparison of concomitant diseases, substance abuse, and internet use in the study subgroups.

	Not Addicted to Internet (*n* = 1722)	Internet Addiction (*n* = 95)
**Concomitant Diseases**		
taking any medication regularly	475 (27.6%)	20 (21.1%)
smoker	**242 (14.1%)**	33 (34.7%) **
taking alcohol	**76 (4.4%)**	17 (17.9%) **
taking drugs	**37 (2.1%)**	15 (15.8%) **
diabetes	**122 (7.1%)**	13 (13.7%) *
hypertension	387 (22.5%)	27 (28.4%)
cardiovascular disease	175 (10.2%)	11 (11.6%)
musculoskeletal pain	136 (7.9%)	10 (10.5%)
history of depression	**19 (1.1%)**	8 (8.4%) **
**Daily Internet Use (Approximately)**		
1 h	684 (39.7%)	12 (12.6%) **
2 h	552 (32.1%)	17 (17.9%) *
3 h	265 (15.4%)	22 (23.2%) *
4 h	114 (6.6%)	18 (18.9%) *
5 h	46 (2.7%)	14 (14.7%) **
6 h	30 (1.7%)	4 (4.2%)
>6 h	31 (1.7%)	8 (8.4%) **
**Daily Time Interval of Internet Use (Multiply Answer)**		
between 0 and 3 a.m.	178 (10.3%)	8 (8.4%)
between 3 and 6 a.m.	69 (4%)	6 (6.3%)
between 6 and 9 a.m.	218 (12.7%)	15 (15.8%)
between 9 and 12 a.m.	335 (19.5%)	14 (14.7%)
12–3 p.m.	196 (11.4%)	13 (13.7%)
3–6 p.m.	410 (23.8%)	31 (32.6%)
6–9 p.m.	894 (51.9%)	49 (51.6%)
9.12 p.m.	332 (19.3%)	25 (26.3%)
**Goal of Internet Use (Multiply Answer)**		
learning/working	1613 (93.7%)	76 (80%) **
internet gaming	**135 (7.8%)**	24 (25.2%) **
chat	**372 (21.6%)**	38 (40) **
community portal (Facebook, Twitter, etc.)	724 (42%)	49 (51.6%)
matchmaking	**41 (2.4%)**	11 (11.6%) **
movies	308 (17.9%)	20 (21%)
music	514 (29.8%)	25 (26.3%)
other	188 (10.9%)	8 (8.4%)

** *p* < 0.001; * *p* < 0.05.

**Table 5:** Risk factors associated with internet addiction in a multivariate analysis.

Risk Factor	OR	CI	*p* Value
age < 35 years	6.098	5.09–7.08	<0.001
male gender	5.413	4.39–6.18	0.002
>5 h daily internet use	2.568	2.03–3.39	<0.001
having no children	1.353	1.13–1.99	0.0248
having secondary employment	11.377	8.67–13.07	0.001

## Data Availability

The dataset supporting the conclusions of this article is available on request to the corresponding author.
